# Development of a Population Pharmacokinetic Model for Cyclosporine from Therapeutic Drug Monitoring Data

**DOI:** 10.1155/2021/3108749

**Published:** 2021-04-08

**Authors:** Martín Umpiérrez, Natalia Guevara, Manuel Ibarra, Pietro Fagiolino, Marta Vázquez, Cecilia Maldonado

**Affiliations:** Pharmaceutical Sciences Department, Faculty of Chemistry, Universidad de la República, Uruguay, General Flores, 2124 Montevideo, Uruguay

## Abstract

**Aim:**

To develop a population pharmacokinetic model for Uruguayan patients under treatment with cyclosporine (CsA) that can be applied to TDM. *Patients and Methods*. A total of 53 patients under treatment with CsA were included. 37 patients with at least one pharmacokinetic profile described with four samples were considered for model building, while the remaining 16 were considered for the assessments of predictive performances. Pharmacokinetic parameter estimation was performed using a nonlinear mixed effect modelling implemented in the Monolix® software (version 2019R1, Lixoft, France); meanwhile, simulations were performed in R v.3.6.0 with the mlxR package.

**Results:**

A two-compartment model with a first-order disposition model including lag time was used as a structural model. The final model was internally validated using prediction corrected visual predictive check (pcVPC) and other graphical diagnostics. A total of 621 CsA steady-state concentrations were analyzed for model development. Population estimates for the absorption constant (ka) and lag time were 0.523 h^−1^ and 0.512 h, respectively; apparent clearance (CL/F) was 30.3 L/h (relative standard error [RSE] ± 8.25%) with an interindividual variability of 39.8% and interoccasion variability of 38.0%; meanwhile, apparent clearance of distribution (Q/F) was 17.0 L/h (RSE ± 12.1%) with and interindividual variability of 53.2%. The covariate analysis identified creatinine clearance (ClCrea) as an individual factor influencing the Cl of CsA. The predictive capacity of the population model was demonstrated to be effective since predictions made for new patients were accurate for C1 and C2 (MPPEs below 50%). Bayesian forecasting improved significantly in the second and third occasions.

**Conclusion:**

A population pharmacokinetic model was developed to reasonably estimate the individual cyclosporine clearance for patients. Hence, it can be utilized to individualize CsA doses for prompt and adequate achievement of target blood concentrations of CsA.

## 1. Introduction

With the approval of the calcineurin inhibitor cyclosporine (CsA), a new era in immunopharmacology began. CsA, a cyclic endecapeptide, has been the cornerstone of most immunosuppressive regimens in organ transplantation. In 1994, the Food and Drug Administration (FDA) approved tacrolimus, another calcineurin inhibitor as an effective alternative to CsA. Several studies have shown that the use of tacrolimus is associated with a lower allograft rejection rate compared with CsA [1–3]. However, CsA is still widely used in clinical practice, predominantly for the prevention of rejection in various types of organ transplantation, to prevent graft-vs.-host disease after bone-marrow transplantation and in a variety of inflammatory and autoimmune diseases such as nephrotic syndrome, Crohn's disease, psoriasis, and focal segmental glomerulosclerosis [4–6].

CsA is extensively metabolized by CYP3A4 and to a lesser extent by CYP3A5 in the liver and in the gastrointestinal tract by CYP3A4, being the content of this enzyme much higher in the intestine than in the liver [7, 8]. CsA is a substrate of P-glycoprotein, and it is transported out of cells via this efflux pump [9]. Both CYP3A4 and Pgp content in the intestine are responsible for its low bioavailability (27%).

Pharmacokinetic parameters of CsA are highly variable and depend on factors such as age, sex, bodyweight, the pathology of the patient, days posttransplantation, comedication, and creatinine clearance among others [10–13]. In addition, CsA has a narrow therapeutic index; thus, therapeutic drug monitoring (TDM) provides a useful tool to individualize therapy minimizing the probabilities of therapeutic failure and toxicity. Trough concentrations (C0) are frequently monitored for this purpose, although poor correlation has been found between this observation and drug mean exposure measured as the area under the concentration versus time curve for the interdose interval at steady state (AUC_T_), which has been recognized as the pharmacokinetic metric that better predicts drug response. Several authors proposed monitoring of 2-hour postdose concentration of CsA (C2) as a better surrogate for CsA AUC_T_ [14–17].

Over the past decade, the precision medicine paradigm has emerged, largely supported by technological developments and advances in artificial intelligence methodologies. Under this approach, different strategies are framed to aim the individualization of medical treatments according to the characteristics of the patient. Genetic information, biomarkers, and phenotypic and psychosocial characteristics are used to feed and develop tools that allow distinguishing patients within a population with similar general clinical conditions and therefore adapt the available therapeutic tools to optimize the clinical outcome at the individual level. The use of computational models to support decision-making related to pharmacotherapy is therefore within the precision medicine paradigm. This approach, which belongs to the discipline of pharmacometrics, has been recently addressed as model-informed precision dosing (MIPD) [18]. The goal is to deliver the right dose, at the right patient, at the right time. Its application in TDM supporting dose optimization of narrow therapeutic index drugs has gained momentum [19], allowing integration of available knowledge in a mathematical model which is implemented to individualize dosing regimens with a Bayesian forecasting framework [20].

Pharmacometric models provide population pharmacokinetic and pharmacodynamic information, estimating parameters for the dose-dependent mean behavior of drug exposition and effect throughout time and quantifying different levels of variability, mainly the between-subject (interindividual) and between-occasion (intraindividual) variabilities. During model development, the effects of individual variables on pharmacokinetic and pharmacodynamic processes are recognized and quantified in a covariate model. This framework offers a suitable tool to select the first dose in a new patient according to specific characteristics. Afterwards, when drug concentrations are observed in that patient, data can be included in the model using the population parameters to describe the prior distribution and estimating individual parameters for further dose optimization [21].

The aim of this work was to develop and implement prospectively in the clinical setting a population pharmacokinetic model for the Uruguayan patients under CsA treatment.

## 2. Material and Methods

### 2.1. Patients and Data Collection

Pharmacokinetic data from CsA TDM routine of patients under treatment either for organ transplantation or for autoimmune diseases was retrospectively analyzed. Data CsA blood concentrations taken from patients with at least one pharmacokinetic profile described with four samples were included in the analysis for model building. This group of patients was considered for the training data set (Group A).

Data including sex, age, bodyweight, medication history, dosage regimen, time of last dose, sampling time, information on concomitant medications, and days posttransplant was collected from the formulary provided by the TDM service. Other relevant information coming from hematological and biochemical tests was obtained from the hospital database system.

After the model was developed and internally evaluated, a prospective evaluation of the predictive capacity of the model, in which the above described inclusion criteria were met plus having at least two occasions of routine CsA blood levels monitored over a period of 18 months, was implemented (Group B).

### 2.2. Measurement of CsA Concentration

Steady-state blood samples were withdrawn at 0, 1, 2, 3, and 4 hours postdosing (C0-C1-C2-C3-C4) and placed into EDTA-containing tubes. Determination of CsA concentrations in whole blood sample was performed using chemiluminescent microparticle immunoassays (CMIA Architect®, Abbot Laboratories). The lower limit of quantification was 12.5 ng/mL, and linearity was proven up to 1500 ng/mL. For concentrations located at lower, middle, and higher portions of the calibration range, coefficients of variation (precision) were 4.1, 2.6, and 0.56%, respectively, and relative errors (accuracy) were 4.1, 8.3, and 6.2%, respectively.

### 2.3. Pharmacokinetic Model of Cyclosporine

The population pharmacokinetic analysis was conducted using a Monolix Suite 2019R1 (Lixoft, France). Briefly, a nonlinear mixed effect model was built through estimation by maximum likelihood using the Stochastic Approximation Expectation Maximization (SAEM) algorithm [22].

Model development was guided with both metrics and graphical diagnostics. Model data fit was assessed with the Akaike information criterion (AIC) and inspection of goodness of fit plots. These included population observations versus model predictions and population residuals and normalized prediction distribution errors (NPDE) [23] versus time and versus the dependent variable; last, TDM data has a large variability in dose regimes, because these prediction-corrected visual predictive checks (pcVPC) were utilized as the main simulation-based diagnostic in model evaluation [24]. In this graphic, both observed and simulated drug concentrations are normalized based on the typical population prediction for the median time in the bin.

The uncertainty of the estimated population parameters was calculated via the estimation of the Fisher Information Matrix in Monolix.

Different mammillary models were evaluated for the disposition of CsA. Drug transference processes between compartments in all cases were assumed to follow first-order kinetics. Secondly, different modeling strategies for the absorption phase were assessed: immediate first-order absorption; lagged first-order absorption; parallel first-order absorption and transit model absorption.

Interindividual variability (IIV) was tested for each parameter as well as interoccasion variability (IOV) assuming an exponential error model as described below:

(1) $$\begin{array}{c} \theta_{i}=\theta \text{pop}*e^{\eta_{i}}, \end{array}$$

where *θ*_*i*_ is the parameter estimate for the subject *i*, *θ*pop the typical value for the population, and *η*_*i*_ the subject discrepancy from *θ* assumed to be normally distributed with mean zero and variance *ω*^2^. IIV and IOV were expressed as coefficients of variation (CV), estimated from the respective variance as

(2) $$\begin{array}{c} \text{CV}=100*\sqrt{e^{\omega^{2}}-1}. \end{array}$$

In the case of IOV, the implementation is analogue, being the gamma of the standard deviation of the interoccasion parameter discrepancies among all patients.

Residual variability was described with a combined error model:

(3) $$\begin{array}{c} Y_{ij}=C_{ij}*\left(1+e,\text{pro}{\text{p}}_{ij}\right)+e,\text{ad}{\text{d}}_{ij}, \end{array}$$

where *Y*_*ij*_ stands for the observed concentration and *C*_*ij*_ denotes the predicted concentration for the subject *i* at time *j* using the pharmacokinetic model described above. Residual error for each observation has therefore an additive (*e*, add_*ij*_) and a proportional (*e*, prop_*ij*_) component which are normally distributed with mean of 0 and variances *σ*, add^2^ and *σ*, prop^2^, respectively.

#### 2.3.1. Covariate Analysis

Covariate analysis was performed by stepwise forward inclusion and backward deletion, assessing the impact of variables with pharmacological and physiological plausibility of having an impact on CsA pharmacokinetics. Initially, univariate likelihood ratio test was performed for each variable, selecting for inclusion in a full model those variables which reduced the objective function value (OFV) by at least 3.84 points. This magnitude corresponds to a 5% type I error for the null hypothesis of one variable having no effect in the model fit, provided that the compared models are nested. Backward deletion was performed from the full model with a 1% type I error, preserving in the final model those covariates for which exclusion increased the OFV in at least 6.63 points.

The impact of continuous and categorical variables over CsA absorption and disposition pharmacokinetic parameters was assessed, including sex, bodyweight, age, comedication, reason of treatment, and creatinine clearance estimated from serum creatinine using the Cockcroft and Gault [25] formula. The following general covariate model was used:

(4) $$\begin{array}{c} {\text{TVP}}_{i}=\theta_{n}\times \overset{m}{\underset{i}{\prod }}{\left(\frac{{\text{Cov}}_{m,i}}{{\text{Cov}}_{\text{ref}}}\right)}^{\beta_{m+n}}\times \overset{p}{\underset{i}{\prod }}\beta_{p+m+n}^{{\text{Cov}}_{p,i}}. \end{array}$$

Typical value of a model parameter (TVP) is described as a function of *m* continuous (Cov_*m*_) and *p* categorical (Cov_*p*_) covariates.

*θ*_*n*_ describes the typical parameter value for and *i*^th^ individual with covariate values (Cov_*m*,*i*_) equal to the reference values: (Cov_*m*,*i*_ = Cov_*m*,ref_) and (Cov_*p*,*i*_ = 0).

Cov_*m*,ref_ refers usually to the median value across the studied population.

*β*_*m*+*n*_ and *β*_*p*+*m*+*n*_ are parameters quantifying the magnitude of the covariate parameter relationship.

#### 2.3.2. Predictive Performance Assessment of the Model

Predictive performance was assessed with patients of Group B as already described above. Simulations were performed in R v.3.6.0 with the mlxR package [26] (Inria Xpop team, v. 3.2). The following procedure was conducted:

1. CsA blood concentrations were simulated for the first occasion of each patient using population pharmacokinetic parameters obtained in the final model, including patient characteristics for the covariate model. Simulated data (*C*_Sim1_) was compared to experimental concentrations (*C*_exp1_) determined by immunoassay analysis
2. Experimental concentrations of occasion 1 were then used to obtain individual pharmacokinetic parameters by maximum a posteriori estimation (MAP), using the population distribution as prior information. This allowed Bayesian forecasting for the CsA whole blood levels in each patient, providing information for dose optimization
3. On the second occasion of each Group B patient, a new experimental CsA concentration was obtained at a specific postdosing time (*C*_exp2_). The individual parameters estimated in step 2 were used to simulate whole blood CsA levels for this second occasion (*C*_Sim2_). Model performance was then assessed on this second occasion by comparing *C*_exp2_ and *C*_Sim2_
4. Experimental concentrations of occasions 1 and 2 were then used to update individual pharmacokinetic parameters by MAP and predict CsA levels to support dose optimization. This process was repeated for subsequent occasions

On each occasion, the relative individual prediction error (rIPE) was calculated for all patients as it is shown in equation (5). The comparison of simulated and experimental concentrations was performed computing the relative bias with the MPPE (Mean Percentage Predictive Error); meanwhile, precision was assessed with the relative Root Mean Squared Prediction (rRMSE) [27]

(5) $$\begin{array}{c} \text{rIPE}=\frac{\left(C_{{\text{Simulated}}_{i}}-C_{{\text{Experimental}}_{i}}\right)}{C_{{\text{Experimental}}_{i}}}\times 100, \end{array}$$

(6) $$\begin{array}{c} \text{MPPE}=\frac{1}{n}\overset{1}{\underset{i}{\sum }}\left[\frac{\left(C_{{\text{Simulated}}_{i}}-C_{{\text{Experimental}}_{i}}\right)}{C_{{\text{Experimental}}_{i}}}\right]\times 100, \end{array}$$

(7) $$\begin{array}{c} \text{rRMSE}=\sqrt{\left\lfloor \frac{1}{n}\overset{i}{\underset{1}{\sum }}\frac{{\left(C_{{\text{Simulated}}_{i}}-C_{{\text{Experimental}}_{i}}\right)}^{2}}{{\left(C_{{\text{Experimental}}_{i}}\right)}^{2}}\right\rfloor }\times 100, \end{array}$$

where *n* represents the number of individuals.

These estimators allowed us to evaluate our model and assess the impact of including new experimental data (priors) to update patient individual parameters, comparing model bias and between successive occasion precision not only to evaluate the predictive performance of our model but also to compare the successive occasions.

## 3. Results

A total of 621 CsA blood concentrations from 37 patients (21 women, 16 men) were included for model building and internal evaluation (training data set). The characteristics of the patients are summarized in Table 1.

**Figure 1:**
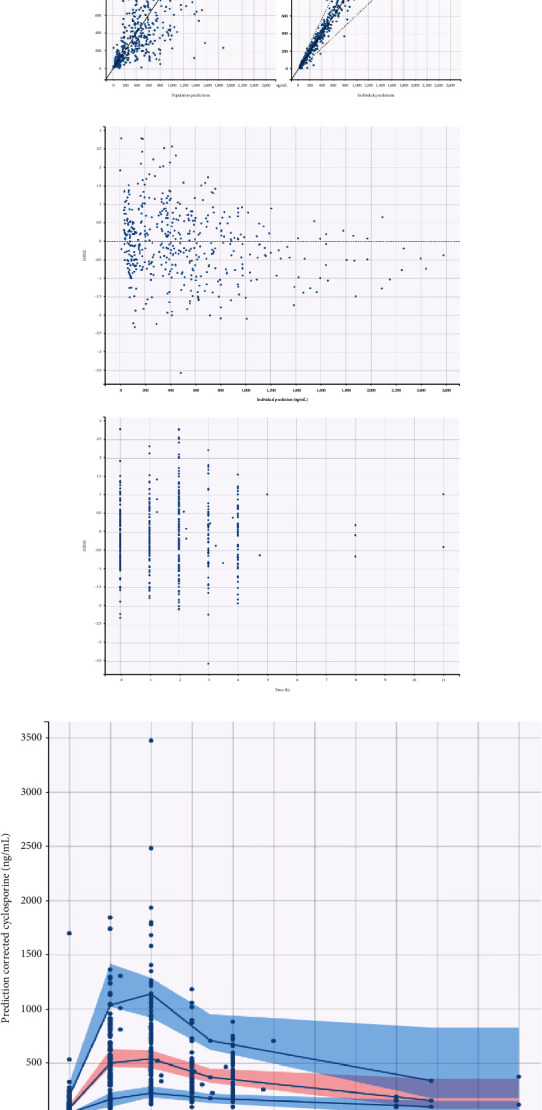
Diagnostic evaluations of the final model for CsA. (a) Observed CsA concentrations vs. population (left plot) and individual (right plot) predictions. (b) Normalized prediction distribution errors (NPDE) vs. time in hours (top plot) and vs. individual prediction CsA concentrations in ng/mL (bottom plot). (c) Prediction corrected visual predictive check (pcVPC) plot for the final model of CsA. Visual predictive check for the final model of CsA shows the observed concentrations (ng/mL) of CsA (circles), the 50th percentile along with the 10th and 90th (blue lines) percentiles of the observed concentration data, and the simulated confidence intervals for each percentile (red and blue shaded areas for the median and 5th and 95th, respectively).

The parameters of the final model estimates are summarized in Table 2. The model that best fits CsA observations (AIC = 6204) was a two-compartment model including lag time, with absorption (ka) and disposition first-order rate constants. The model was parametrized in terms of ka, tlag (latency time for drug absorption), apparent clearance (Cl/F), apparent clearance of distribution (Q/F), apparent volumes of distribution for the central (V1), and peripheral (V2) compartments. Goodness of fit plots and metrics for the final model are shown in Figure 1.

In accordance with previous reports, we found high intra- and intervariability for pharmacokinetic parameters of CsA. For instance, Cl/F showed a high variability both between subjects (IIV = 39.8%) and between occasions (IOV = 38.0%).

Age, sex, bodyweight, comedication, and reason of treatment were not identified as significant covariates for CsA clearance. This model was constructed based on retrospective data, and even though hematocrit has been lately related as a covariate affecting CsA population pharmacokinetic models, this information was not available in clinical charts, so it was not included. Only creatinine clearance showed to be a significant covariate for CsA clearance in the PK model. The value of creatinine clearance can be interpreted as a renal function evaluator. The result obtained for *β*_ClCrea_ was -0.204, considering that individual Cl can be calculated as

(8) $$\begin{array}{c} {\text{Cl}}_{i}={\text{Cl}}_{\text{pop}}*{\left(\frac{{\text{ClCrea}}_{i}}{{\text{ClCrea}}_{\text{pop}}}\right)}^{\beta_{\text{ClCrea}}}, \end{array}$$

where Cl_*i*_ is the individual clearance of CsA, Cl_pop_ is the population clearance determined in the PK model, ClCrea is the individual value of creatinine clearance, ClCrea_pop_ is the mean value for creatinine clearance (98.62 mL/min), and *β*_ClCrea_ represents how this covariate impacts on the parameter.

The predictive performance data set was comprised by 81 CsA whole blood concentrations from 16 patients included in Group B (10 women, 6 men). The characteristics of the patients are summarized in Table 1 as well.

## 4. Discussion

In this study, a population pharmacokinetic model of CsA was developed and its predictive performance tested, evaluating its routine implementation within the TDM service to make corresponding dose adjustments, therefore maximizing the probability of achieving effectiveness and safety in CsA treatments.

Creatinine clearance was the only final significant covariate in the model, showing a negative correlation with CsA apparent clearance, i.e., a lower creatinine clearance is correlated with an increase in CsA apparent clearance. Quantification of this covariate effect is of particular importance to quantify CsA elimination clearance under renal impairment and make proper dose adjustments. Previous studies carried out by our research group on the impact of cardiovascular physiology in pharmacokinetic processes provide background to understand this effect, which we believe affects CsA disposition [28, 29]. In fact, this relationship was previously described by Eiraldi et al. [30]. Although CsA shows hepatic and intestinal metabolism, its elimination clearance can be affected in renally impaired patients because of a change in the relative blood flow fractions being delivered to the different organs. When the renal blood flow is affected, which can happen because of CsA induced toxicity or CsA subtherapeutic levels in renal transplanted patients, the fraction of blood flow directed to the splanchnic region will increase, therefore increasing CsA systemic clearance. This covariable was preferred to postoperative days as it is considered that both parameters share predictive and mechanistic information on CsA clearance. Short after transplant, renal blood flow is negligible and creatinine levels high; however, if transplantation is successful, as hours go by, the organism accepts the graft and the creatinine level normalizes, reducing blood flow to the splanchnic region and therefore CsA clearance. Nevertheless, grafts maintain acceptable function for a period and functionality decreases with years and so does creatinine clearance. Therefore, for higher POD creatinine clearance decreases whereas CsA clearance increases due to splanchnic elimination.

**Figure 2:**
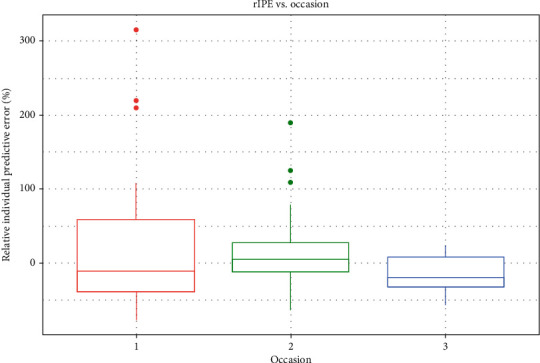
Boxplots of relative individual predictive error (rIPE, %) vs. occasion. The amount of observations on each occasion was indicated in Table 1. In this scenario, occasion 1 represents the prediction without prior information, and then 2 and 3 represent the prediction with 1 and 2 prior observations, respectively.

Model implementation in the clinical setting showed interesting results. Predictions made for new patients using only the population pharmacokinetic model and the individual creatinine clearance value were reasonably accurate for C1 and C2, with MPPEs below 50%, which are not too high if we consider CsA pharmacokinetic variability (both inter- and intraindividual). The estimation of C0 gave poor results for this first occasion. However, this improved remarkably after the inclusion of patient data, reducing the MPPE from 98% to 27% at the third occasion. Accuracy indicators for C1 also improved significantly for the second and third occasions, whereas the prediction of C2 improved also for the second and third occasions. Prediction improvement is shown in Figure 2 as a boxplot of the rIPE vs. prior information. Overall, the estimation of individual parameters using very sparse patient data and the population distribution (the model) are shown to significantly increase the accuracy of model predictions. Considering again the high pharmacokinetic variability that CsA shows, the use of this approach provides a powerful tool to perform dose optimization in the clinical setting.

Some limitations of the study should be addressed. In our data, the reason of treatment (renal transplantation-autoimmune disease) was analyzed as a covariate resulting in nonsignificance on any of the pharmacokinetic parameters; however, these results may not be conclusive as the number of subjects on each group was small. The lack of multiple validation cohorts is a limitation for the extrapolation of model predictions in other populations; therefore, the reported CYA population pharmacokinetic model should be externally evaluated with target population before implementation in other centers.

## 5. Conclusions

A population pharmacokinetic model of CsA was developed and implemented to predict CsA whole blood concentrations in the clinical setting for patients under different dosage regimens and pathologies. Creatinine clearance was shown to be the only significant covariate able to partially explain the interindividual variability observed in CsA apparent clearance. The model showed a good predictive performance, which significantly improved with the inclusion of individual patient data and Bayesian forecasting, standing out as a valuable tool to support dose optimization.

## Figures and Tables

**Table 1:** 

	Group A	Group B
Total number of patients	37	16
Number of observations	621	81
Sex (male/female)	16/21	6/10
Age (mean ± SD, years)	34.4 ± 15.85	39.6 ± 19.4
BW (mean ± SD, kg)	64.3 ± 11.0	63.7 ± 11.1
Serum Cr (mean, mg/dL)	1.11 (5.9-0.2)	1.71 (7.71-0.36)
Cl Cr (mean, mL/min)	98.62 (417.92-13.79)	98.56 (240.05-7.71)
Reason of treatment	Kidney transplant (14) Liver autoimmune diseases (1) Bone marrow transplant (1) Kidney autoimmune diseases (21)	Kidney autoimmune diseases (8) Bone marrow transplant (2) Kidney transplant (5) Erythroblastopenia (1)

**Table 2:** 

Parameter	Mean	RSE (%)	Description
Tlag (h)	0.512	8.48	Latency time
Ka (h^−1^)	0.523	8.54	Absorption constant
Cl/F (L/h)	30.3	8.25	Apparent clearance
*β*_CL-CLCr_	-0.204	43.7	$\text{loglog }\left(\text{CLi}\right)=\text{loglog }\left(\text{CLpop}\right)+\beta_{\text{CL}-\text{CLCr}}*\log\;\left(\frac{\text{CLCri}}{\text{CLCr}}\right)+\eta_{\text{CL}}\;$
Q/F (L/h)	17.0	12.1	Apparent clearance of distribution
V1 (L)	17.9	17.6	Apparent volume of central compartment
V2 (L)	400	45.6	Apparent volume of peripheral compartment
IIV Cl (%)	39.8	16.6	Interindividual variability of Cl
IIV Q (%)	53.2	18.7	Interindividual variability of Q
IOV Cl (%)	38.0	8.53	Interoccasion variability of Cl
IOV tlag (%)	54.1	12.4	Interoccasion variability of tlag
IOV ka (%)	52.6	9.85	Interoccasion variability of ka
Ka-Cl	-0.551	20.2	Correlation between Ka-Cl
Prop (%)	0.228	8.86	Proportional error
Add (ng/mL)	7.52	45.0	Additive error
AIC	6204		Akaike information criterion

## Data Availability

The data used to support the findings of this study are available from the corresponding author upon request.
